# Comparison of Imaging Severity Between Vaccinated and Unvaccinated COVID-19 Patients: Perspective of an Indian District

**DOI:** 10.7759/cureus.30724

**Published:** 2022-10-26

**Authors:** Joy Singhal, Chetan Goel, Vinit Gupta, Mandeep Sachdeva, Shaurya Sanjappa, Vipin Koushal, Inderpreet Singh, Akash Tripathi

**Affiliations:** 1 Respiratory Medicine, District TB (Tuberculosis) Hospital, Ambala, IND; 2 Cardiology, Grange University Hospital, Newport, GBR; 3 Radiodiagnosis, Unispital Healthcare, Ambala, IND; 4 Hospital Administration, Postgraduate Institute of Medical Education and Research, Chandigarh, IND; 5 Internal Medicine, West Suffolk Hospital, Bury St Edmunds, GBR; 6 Emergency, Manipal Hospital, Patiala, IND; 7 Stroke Medicine, Stepping Hill Hospital, Manchester, GBR; 8 Family and Community Medicine, CHC (Community Health Centre) Bungal Badhani, Pathankot, IND

**Keywords:** hrct chest (high resolution computed tomography), covid-19 vaccine, computed tomography severity score (ctss) of chest, severe covid-19, covid-19

## Abstract

Background: Extensive vaccination drives undertaken globally helped in the fight against the coronavirus disease 2019 (COVID-19) pandemic, but different nations adopted different vaccination policies to tackle the disease. The vaccination drive in India began with the administration of two different vaccines: Covishield and Covaxin. We assessed the effect of vaccination status on imaging severity in patients with positive COVID-19 reverse transcription-polymerase chain reaction (RT-PCR)/antigen tests.

Method: This was a single-center retrospective observation analysis carried out over three months between March 1, 2021, to May 31, 2021. Data access was provided by the District Hospital Review Board (DHRB) and the Department of Health (DOH), District Ambala, Haryana. Appropriate statistical tools were used to analyze the data. Statistical Package for Social Sciences (SPSS) 26.0 and Python 3.9 were used for statistical analysis and visualization, and a p-value of less than 0.05 was considered statistically significant.

Results: The total sample size of the study was 1,316, out of which 371 (28.2%) were vaccinated and 945 (71.8%) were not vaccinated. The mean age of the study participants was 49.6 ± 15.7 years. Seven hundred ninety-seven (60.6%) participants were male, while 519 (39.4%) participants were female. A statistically significant reduction was observed in the computed tomography severity score (CTSS) of the vaccinated population compared to the non-vaccinated group (χ^2^ = 74.3, p < 0.001). Vaccination led to a statistically significant decrease in mean CTSS across all lung lobes.

Conclusion: Emerging COVID-19 variants challenge the effect of available vaccines, with different nations adopting different vaccination strategies to deal with the ongoing health problem. CTSS was employed as an objective marker to study the disease severity and effect of vaccination. Vaccination resulted in a significant reduction in CTSS seen on high-resolution computed tomography (HRCT) chest scans. There was a significant decrease in the incidence of severe COVID-19 pneumonia among vaccinated individuals. We need more observational data to corroborate the efficacy of vaccines presented in the randomized trials. Sharing such data between different nations can help us adopt a unifying vaccination strategy and decrease the impact of COVID-19 in subsequent disease waves.

## Introduction

The coronavirus disease 2019 (COVID-19) pandemic is still raging after its first outbreak in Wuhan in 2019, with new infection waves that keep emerging with different variants that keep challenging the efficacy of developed vaccines and diagnostic tests [1]. Extensive vaccination drives undertaken all over the world helped to fight the pandemic, but the population coverage remained highly variable with each nation adopting a different vaccination policy and using a different vaccine for its population subject to availability. There were two vaccines approved in India at the time of the study: Covishield and Covaxin both of which required two doses for full vaccination. There has been a developing body of evidence that has shown that emerging variants can evade the action of neutralizing antibodies, which may cause a decline in vaccine immunity over time and lead to a rise in the incidence of breakthrough infections [2]. There is emerging evidence to support the use of a booster dose after a full vaccination course, which is being adopted by various nations to reduce the severity of symptoms and critical outcomes for hospitalized patients [3]. Data supporting the immunity developed due to previous severe acute respiratory syndrome coronavirus-2 (SARS‑CoV‑2) in reducing subsequent infections or disease severity are still unclear, especially under the pretext of patients who were further vaccinated after having the infection.

Assessment of disease severity requires objective quantitative tools to reduce intrapatient variability encountered using qualitative tools. Patients with COVID-19 benefit from chest computed tomography (CT) imaging for screening and dynamic evaluation [4]. Several scores have been proposed to assess the severity of pulmonary parenchymal involvement in different lung infections. We used computed tomography severity score (CTSS) to study lung involvement in COVID-19 patients and assess the relationship with their vaccination status. We employed CTSS as it enables a quick assessment of pulmonary affection and had demonstrated excellent results in predicting severe COVID-19 infection [5]. This study aims at comparing the CTSS between the vaccinated and the unvaccinated COVID-19 patients assessed using a chest high-resolution computed tomography (HRCT) scan.

## Materials and methods

Study design

This is a single-center observational retrospective analysis that was conducted for three months in adult patients diagnosed with COVID-19. We indexed all COVID-19 patients over the age of 18 years in the Government District Hospital, Ambala, Haryana. CTSS was employed in assessing the extent of lung involvement and as an objective parameter to mark disease severity [4]. Appropriate statistical tools were then used to review the patient data and assess the effect of the vaccine (Covishield: a chimpanzee adenoviral vectored vaccine with full-length SARS-CoV-2 spike insert developed at the University of Oxford (Oxford, UK) and manufactured by the Serum Institute of India, and Covaxin: a whole inactivated virus-based COVID- 19 vaccine developed by Bharat Biotech in collaboration with the Indian Council of Medical Research, National Institute of Virology) in reducing the extent of lung involvement as seen on chest HRCT [6,7].

Data collection

Data were collected between March 1, 2021, and May 31, 2021, from all patients aged over 18 years who had a confirmed positive SARS-CoV-2 reverse transcription polymerase chain reaction (RT- PCR)/antigen test and received a high-resolution CT thorax scan to determine the extent of their lung involvement [4,8]. A total of 2,791 cases were scanned, but only those with positive RT-PCR/antigen tests were included. Cases with missing values were dropped and were not included in the study.

Sample considerations

The vaccination drive was carried out by the Department of Health (DOH), Ambala, across all the villages in the district. The study was conducted during the second vaccination drive in India where only people over the age of 60 or people over the age of 45 with comorbidities were included. Although people between the age group of 18 and 45 years were not part of the vaccination drive, all the healthcare and other frontline workers who were at risk to acquire the COVID-19 infection were vaccinated. Everyone in the population who was eligible had equal access to the vaccine, thereby eliminating any bias in vaccination due to a patient's socioeconomic position, gender, or geographic location. Furthermore, free HRCT scans provided by the government under various schemes ensured that no patient is excluded because of their socioeconomic status, eliminating sampling bias. Vaccination status was confirmed in line with the data maintained online (https://www.cowin.gov.in/) by the Ministry of Health and Family Welfare and National Health Authority of India, accessible across all the healthcare centers in the country. The vaccination status of each individual was linked to their national ID (Aadhaar) accessible online. All individuals who received even a single dose of vaccine were included in the vaccinated group. All individuals with unknown vaccination status were considered unvaccinated.

Imaging analysis

All HRCT thorax scans were performed using a VCT Philips 16 scanner (Philips, Amsterdam, Netherlands). Patients were positioned supine with a single breath hold. The following scanning parameters were used: scan direction (craniocaudally), tube voltage (120 kV), tube current (100-600 mA), smart mA dose modulation, slice collimation (16 × 0.8 mm), width (0.625 mm), pitch (2), rotation time (0.8 s), and scan length (60.00-1300.00 s). The results of the chest HRCT images were collected and analyzed using Picture Archiving and Communication Systems (PACS).

Imaging interpretation

Three radiologists, each with more than eight years of experience, reviewed the images to determine the CTSS in each patient. The scans were first evaluated to determine whether they were negative or positive for typical COVID-19 pneumonia findings (bilateral, multi-lobe, posterior peripheral ground-glass opacities) as defined by the Radiological Society of North America (RSNA) consensus statement [8]. The severity was then determined using the CTSS scoring system, which is based on a visual assessment of each lobe involved [4]. Radiologists were blind to the vaccination status of the patients when determining the severity score.

Statistical analysis

Statistical Package for Social Sciences (SPSS) 26.0 (IBM, New York, USA) and Python 3.9 (Python Software Foundation, Delaware, USA) with relevant libraries were used for statistical analysis and visualization, and a p-value of less than 0.05 was considered statistically significant. Mean, median, skew, and percentages were calculated for descriptive statistics. The Spearman coefficient was used to measure correlation. χ^2^ test, T-test, and permutation test were used to test for the null hypothesis. A multiple linear regression model was constructed to observe the effects of age, gender, and vaccination status on the CTSS.

## Results

The total sample size of the study was 1,316, out of which 371 (28.2%) were vaccinated and 945 (71.8%) were not vaccinated. The mean age of the study participants was 49.6 ± 15.7 years. There was a statistically significant difference between the mean age of the vaccinated and the unvaccinated group (t-statistic: 8.8, p < 0.05), which could have been due to the preferential vaccination in the higher age groups. Keeping the gender and the vaccination status constant, multiple linear regression analysis showed that increased age was associated with an increased CTSS, with approximately every one-year increase in age leading to a 0.14 increase in CTSS. Seven hundred ninety-seven (60.6%) participants were male, while 519 (39.4%) participants were female. The groupwise distribution of demographic data between the vaccinated and the unvaccinated study groups is presented in Table 1. 

**Table 1:** Groupwise distribution of demographic data between vaccinated and unvaccinated study groups CTSS: computed tomography severity score.

Parameters	Vaccination Status		p-Value
	Non-Vaccinated (N = 945)	Vaccinated (N = 371)	
Age			<0.05
Mean	47.2 ± 15.9	55.6 ± 14.1	
Median	47	58	
Skew	0.14	-0.29	
Gender			>0.05
Male	567 (71.1%)	230 (28.9%)	
Female	378 (72.8%)	141 (27.2%)	
Severity (CTSS Range)			<0.001
Normal (0)	31 (3.28%)	60 (16.17%)	
Mild (1-10)	287 (30.37%)	101 (27.23%)	
Moderate (11-20)	316 (33.44%)	126 (33.96%)	
Severe (20-35)	311 (32.91%)	84 (22.64)	

**Figure 1:**
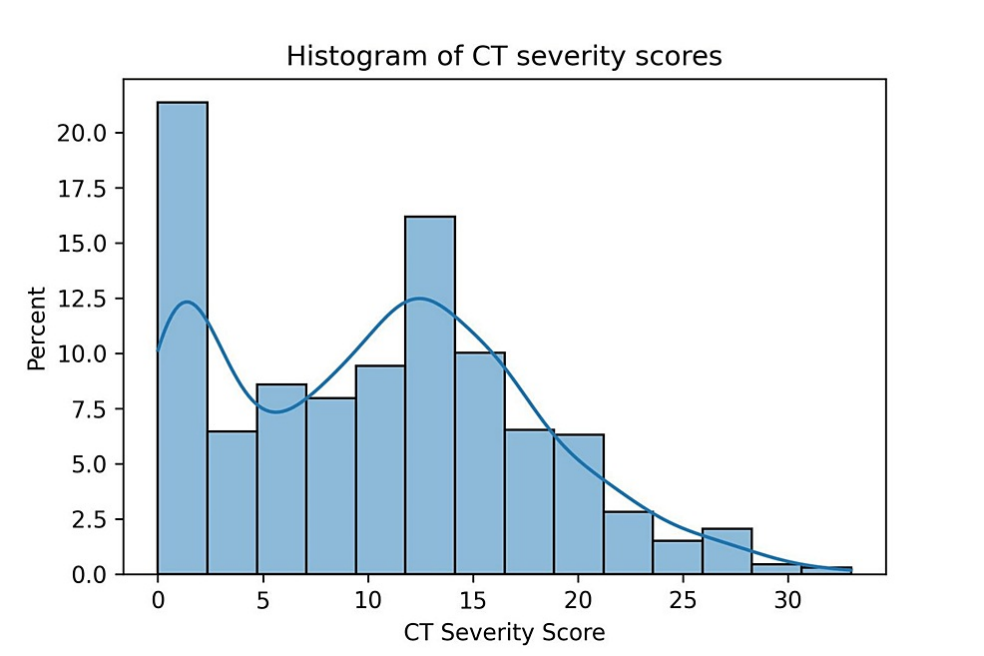
Bimodal distribution of CT severity score in study population CT: computed tomography.

No statistically significant difference was observed between gender and CTSS. A statistically significant difference was observed between the CTSS of the vaccinated and the non-vaccinated groups (χ^2^ = 74.3, p < 0.001). The CT severity scores did not follow a normal distribution (as shown in Figure 1), and hence non-parametric tests were also used to measure the effect of vaccination on CT severity scores. As we could not assume normality, the Spearman coefficient was used to assess the correlation between age and CTSS. There was a positive correlation observed between age and CT severity scores with the Spearman coefficient being 0.21 (p < 0.001). The Mann-Whitney U test showed a statistically significant (p < 0.001) difference between the distribution of CT severity scores of vaccinated and non-vaccinated populations. A T-test was also conducted on the data and showed similar statistically significant results (p < 0.001).

**Figure 2:**
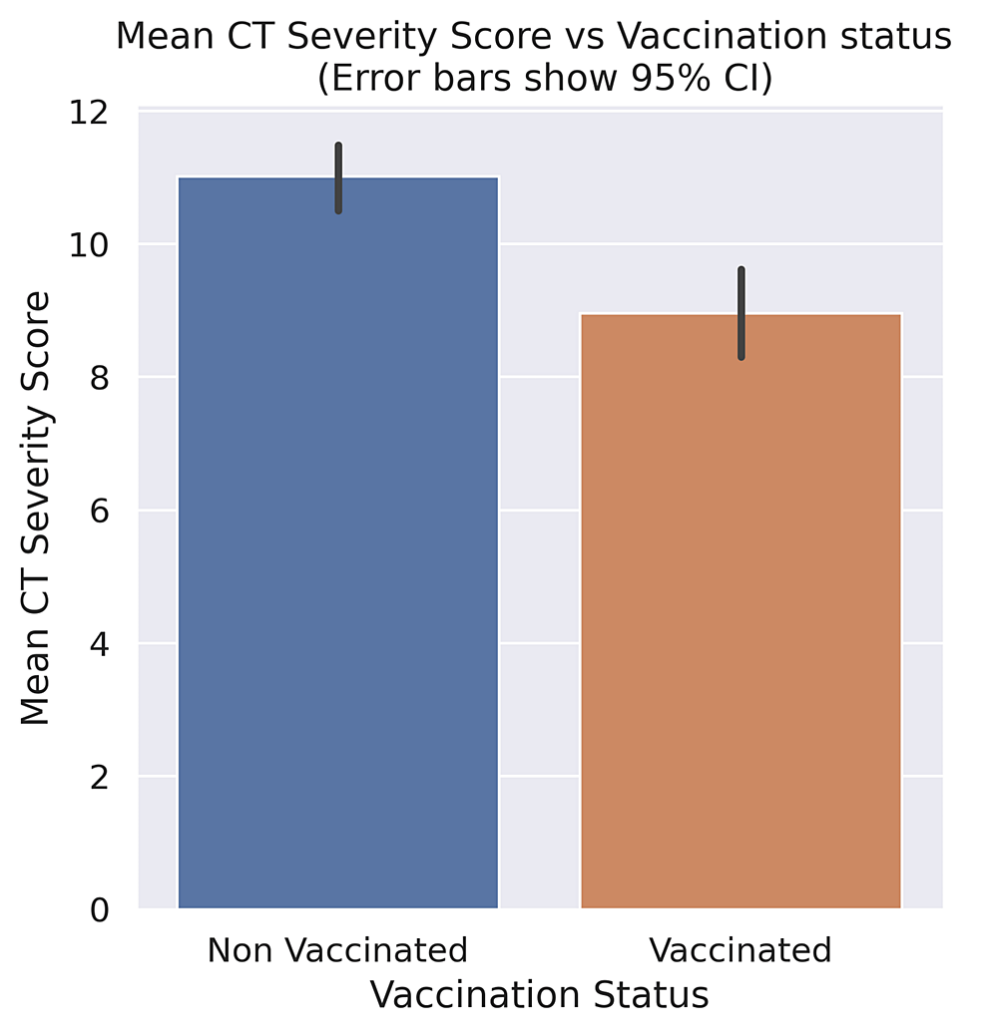
Mean CTSS in vaccinated and unvaccinated study groups CT: computed tomography, CI: confidence interval, CTSS: computed tomography severity score.

A permutation test was used to quantify the difference in CTSS between the two groups. Two stratified samples with resampling were taken from our dataset (the stratification was done based on age and gender). We hypothesized that the mean CT severity score of the vaccinated study group would be greater than that of the non-vaccinated study group. The difference in the mean CT severity score between the vaccinated population and the non-vaccinated population was taken to be the test statistic. This whole process was then repeated 1,000,000 times. The test statistic calculated was -2.06. The test statistic is negative suggesting that the mean CT severity score of the vaccinated group is lower than that of the non-vaccinated group (Figure 2). The probability of obtaining a test statistic greater than or equal to the observed value under the null hypothesis is less than 0.1% (one-sided p < 0.001). This is less than our chosen threshold of 5%, so we consider this to be significant evidence against the null hypothesis.

**Figure 3:**
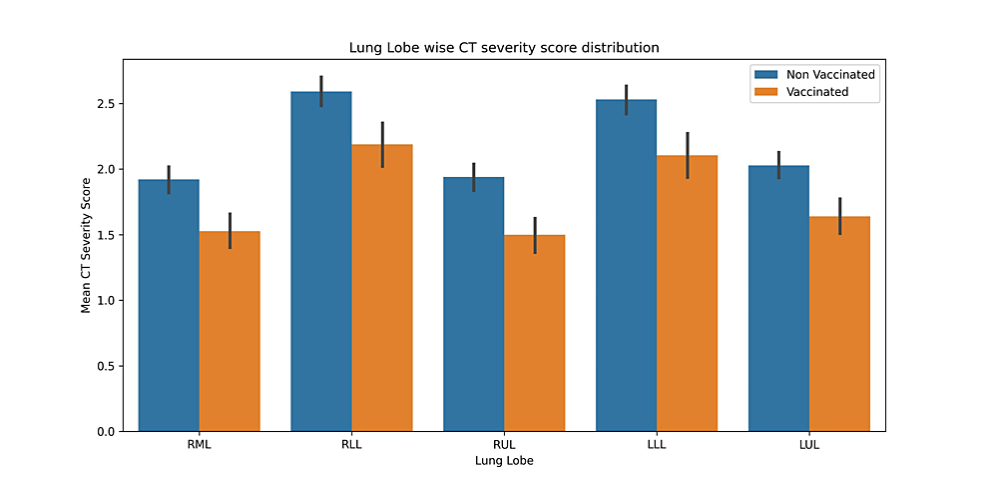
CTSS for different lung lobes in vaccinated and unvaccinated study groups CT: computed tomography, RML: right middle lobe, RLL: right lower lobe, RUL: right upper lobe, LLL: left lower lobe, LUL: left upper lobe.

Vaccination led to a statistically significant decrease in mean CTSS scores across all lung lobes (Figure 3). Our data suggest that the lower lung lobes were more affected compared to the upper lung lobes.

## Discussion

In this study, we assessed the effect of vaccination status on imaging severity in chest HRCT scans among COVID-19 patients as a single-center retrospective observational analysis. Here, we used CTSS as an imaging marker to see the degree of lung involvement in patients with COVID-19 pneumonia. Chest CTSS acts as a more robust and objective parameter to measure organic disease burden in the lungs compared to nonspecific inflammatory biomarkers helping in diagnosis, monitoring treatment, and prognostication. Devised by Yang et al., CTSS is calculated by dividing the lung parenchyma into 20 regions and scoring 0, 1, or 2 for each region based on the degree of opacification, thereby making the score range between 0 and 40 [4]. Multiple studies have been conducted to establish the relationship between CTSS and the severity of COVID-19 pneumonia. Zayed et al. validated the CTSS on par with COVID-19 Reporting and Data System (CO-RADS) scoring in predicting disease severity among COVID-19 patients, with a 90% negative predictive value (NPV) for CTSS to rule out severe COVID-19 compared to 97% NPV for CO-RADS score [5].

COVID-19 vaccination drive began in India on January 16, 2021. India initially approved Covishield (Oxford-AstraZeneca vaccine) and Covaxin (BBV152 developed by Bharat Biotech) for use, whose data have been used in this study. During the duration of data collection for this study, the supply of vaccines went to government-run clinics and was offered free of charge to people aged 45 years and over. The primary objective of COVID-19 vaccines is to prevent severe disease rather than infection [9]. Multiple studies have evaluated the short-term efficacy of available vaccines in achieving the said objective, but data about the long-term effect are still evolving. WHO data suggest that the AstraZeneca vaccine (Cambridge Biomedical Campus, Cambridge, England) has an efficacy of 72% against symptomatic SARS-CoV-2 infection irrespective of the interdose interval from participants receiving two standard doses with intervals varying between four and 12 weeks [10]. Covaxin was shown to have an efficacy of 78% against COVID-19 of any severity and 93% against severe disease [11]. These data corroborate our study finding which suggests a significant reduction in mean CTSS in vaccinated individuals compared to unvaccinated. According to a study published by Watson et al. in the *Lancet*, COVID-19 vaccination prevented additional 19.8 million deaths globally by the end of the first year of vaccine rollout [12]. The long-term effect of boosters on reducing the rate of infections and hospital admissions is largely unknown. We are still evaluating the real-world performance of these vaccines compared to the proclaimed efficacy in randomized trials in achieving these endpoints in different settings, and thus observational studies like ours hold value in providing substantiating evidence.

The protective effect of the vaccine was evident from our study as only 3.28% of the non-vaccinated individuals had a normal CT study compared to the vaccinated group where 16.17% of the patients had a normal study. This could have been one of the biggest contributors to the decrease in mean CTSS. The proportion of severe COVID-19 infection fell from 32.9% in the unvaccinated group to 22.6% in the vaccinated group demonstrating a significant impact in reducing the burden of severe disease. A similar significant difference in the mild and moderately severe COVID-19 cases was not observed. More studies are required to test these observations.

Although no statistical relationship has been observed between CTSS and gender, numerous studies show a significantly higher incidence of severe COVID-19 and mortality due to COVID-19 infection in males than in females [13]. The higher COVID-19 case fatality rate and increased severity of disease in males compared to females are likely due to a combination of behavioral/lifestyle risk factors, the prevalence of comorbidities, aging, and underlying biological sex differences [14]. Several comorbidities, which disproportionally occur in men, likely contribute to worse COVID-19 outcomes. A notable example is that of the immune response: although females generally have an overall stronger immune response, males are more likely to develop the cytokine storm associated with poor COVID-19 outcomes [15]. Although these studies show increased predilection of the male sex for severe COVID-19 infection, this has not been reflected in our study.

A positive correlation was observed between age and CT severity scores in our study. This observation was consistent with other studies regarding increasing age being associated with increased severity of COVID-19 [13,16-19]. The reason for this can be multifactorial. One major factor can be the increased prevalence of other comorbidities in the older age group [20]. Another possible bias could be atypical presentations of diseases in the older age group which leads to delayed diagnosis leading to late presentation to the hospital after considerable disease progression.

This study has a few limitations. The interval between the time of diagnosis and the time of imaging is different for each patient. Thus, the issue of time of imaging and their related implications on the CTSS could not be addressed. We did not have any data about patient comorbidities. Any effect of comorbidities affecting the imaging severity could not be seen. The relationship between imaging severity and disease outcome was not examined. We did not have any data related to several potential confounders like ethnicity, prior or current treatment with steroids, old pulmonary sequelae that could potentially modify the CTSS, prior infections from SARS-CoV-2, and weight or use of preventive measures like masks. Further research is needed to validate the findings of this study and establish the use of CTSS in assessing the effect of vaccination.

## Conclusions

Emerging COVID-19 variants challenge the effect of available vaccines, with different nations adopting different vaccination strategies to deal with the ongoing health problem. CTSS was employed as an objective marker to study the disease severity and effect of vaccination. Vaccination resulted in a significant reduction in CTSS seen on HRCT chest scans. There was a significant decrease in the incidence of severe COVID-19 pneumonia among vaccinated individuals. We need more observational data to corroborate the efficacy of vaccines presented in the randomized trials. Sharing such data between different nations can help us adopt a unifying vaccination strategy and decrease the impact of COVID-19 in subsequent disease waves.

## References

[1] Zhou W, Wang W (2021). Fast-spreading SARS-CoV-2 variants: challenges to and new design strategies of COVID-19 vaccines. Signal Transduct Target Ther.

[2] Garcia-Beltran WF, Lam EC, St Denis K (2021). Multiple SARS-CoV-2 variants escape neutralization by vaccine-induced humoral immunity. Cell.

[3] Chenchula S, Karunakaran P, Sharma S, Chavan M (2022). Current evidence on efficacy of COVID-19 booster dose vaccination against the Omicron variant: a systematic review. J Med Virol.

[4] Yang R, Li X, Liu H (2020). Chest CT severity score: an imaging tool for assessing severe COVID-19. Radiol Cardiothorac Imaging.

[5] Zayed NE, Bessar MA, Lutfy S (2021). CO-RADS versus CT-SS scores in predicting severe COVID-19 patients: retrospective comparative study. Egypt J Bronchol.

[6] (2022). Serum Institute of India: COVISHIELD FAQs. https://www.seruminstitute.com/health_faq_covishield.php.

[7] (2022). Bharat Biotech: COVAXIN: India's first indigenous Covid-19 vaccine. https://www.bharatbiotech.com/covaxin.html.

[8] Simpson S, Kay FU, Abbara S (2020). Radiological Society of North America expert consensus document on reporting chest CT findings related to COVID-19: endorsed by the Society of Thoracic Radiology, the American College of Radiology, and RSNA. Radiol Cardiothorac Imaging.

[9] Scott J, Richterman A, Cevik M (2021). Covid-19 vaccination: evidence of waning immunity is overstated. BMJ.

[10] (2022). World Health Organization: The Oxford/AstraZeneca (chadox1-S [recombinant] vaccine) COVID-19 vaccine: what you need to know. https://www.who.int/news-room/feature-stories/detail/the-oxford-astrazeneca-covid-19-vaccine-what-you-need-to-know.

[11] (2022). World Health Organization: The Bharat Biotech BBV152 COVAXIN vaccine against COVID-19: what you need to know. https://www.who.int/news-room/feature-stories/detail/the-bharat-biotech-bbv152-covaxin-vaccine-against-covid-19-what-you-need-to-know#:~:text=The%20vaccine%20can%20be%20administered,not%20need%20to%20be%20repeated..

[12] Watson OJ, Barnsley G, Toor J, Hogan AB, Winskill P, Ghani AC (2022). Global impact of the first year of COVID-19 vaccination: a mathematical modelling study. Lancet Infect Dis.

[13] Jin JM, Bai P, He W (2020). Gender differences in patients with COVID-19: focus on severity and mortality. Front Public Health.

[14] Bwire GM (2020). Coronavirus: why men are more vulnerable to Covid-19 than women?. SN Compr Clin Med.

[15] Qi S, Ngwa C, Morales Scheihing DA (2021). Sex differences in the immune response to acute COVID-19 respiratory tract infection. Biol Sex Differ.

[16] Zheng Z, Peng F, Xu B (2020). Risk factors of critical & mortal COVID-19 cases: a systematic literature review and meta-analysis. J Infect.

[17] Statsenko Y, Al Zahmi F, Habuza T (2022). Impact of age and sex on COVID-19 severity assessed from radiologic and clinical findings. Front Cell Infect Microbiol.

[18] Romero Starke K, Reissig D, Petereit-Haack G, Schmauder S, Nienhaus A, Seidler A (2021). The isolated effect of age on the risk of COVID-19 severe outcomes: a systematic review with meta-analysis. BMJ Glob Health.

[19] Levin AT, Hanage WP, Owusu-Boaitey N, Cochran KB, Walsh SP, Meyerowitz-Katz G (2020). Assessing the age specificity of infection fatality rates for COVID-19: systematic review, meta-analysis, and public policy implications. Eur J Epidemiol.

[20] Treskova-Schwarzbach M, Haas L, Reda S (2021). Pre-existing health conditions and severe COVID-19 outcomes: an umbrella review approach and meta-analysis of global evidence. BMC Med.

